# LINC01101 and LINC00277 expression levels as novel factors in HPV‐induced cervical neoplasia

**DOI:** 10.1111/jcmm.13288

**Published:** 2017-08-02

**Authors:** Iulia Virginia Iancu, Gabriela Anton, Anca Botezatu, Irina Huica, Anca Nastase, Demetra Gabriela Socolov, Anca Daniela Stanescu, Simona Olimpia Dima, Nicolae Bacalbasa, Adriana Plesa

**Affiliations:** ^1^ Molecular Virology Laboratory Romanian Academy “Stefan S. Nicolau” Virology Institute Bucharest Romania; ^2^ Fundeni Clinical Institute Bucharest Romania; ^3^ “Gr. T. Popa” University of Medicine Iassy Romania; ^4^ Bucur Hospital Bucharest Romania; ^5^ “Carol Davila” University of Medicine and Pharmacy Bucharest Romania

**Keywords:** Cervical cancer, HPV, epigenetic factors, long intergenic non‐coding RNAs (lincRNAs)

## Abstract

Recently long non‐coding RNAs were identified as new factors involved in gene expression regulation. To gain insight into expression pattern of these factors related to E7 HPV18 oncogene, this study uses HeLa cell culture transfected with E7‐siRNA. Gene expression profile was investigated using microarray analysis. After analysing the microarray results, we identified 15,387 RNA species differentially expressed in E7‐siRNA‐transfected cells compared with controls (fold change >2). The expression profiles of lncRNA species highlighted 731 lncRNAs and 203 lincRNAs. We selected two lincRNAs (LINC01101 and LINC00277) and we evaluated the expression profile in HPV‐induced neoplasia. Both lincRNAs investigated display a significantly reduced pattern of expression in cervical lesions and cancer, associated with clinical parameters. A connection between HPV presence and lincRNAs was noted. hrHPV‐positive samples exhibit significantly reduced LINC01101 and LINC00277 expression level (*P* < 0.05). These results provide new insights into involvement of lncRNA in HPV‐induced cervical cancer, enriching our understanding of their potential role in this pathology.

## Introduction

Cervical cancer, responsible for approximately 266,000 deaths each year, is the fourth most frequently type of cancer in women especially in developing countries 1. Epidemiological data established that persistent infections with high‐risk genotypes of human papillomavirus (hrHPV) (such as HPV18 and HPV16) could lead to cervical dysplasia and cancer, thus confirming HPV as an aetiological agent of this malignancy. The mechanism by which hrHPVs contribute to cervical oncogenesis is represented by the action of the two main oncogenes E6 and E7 that target and silence multiple key cellular proteins among the most important being p53 and pRb tumour suppressors that ultimately give rise to cell cycle deregulation and malignant transformation 2.

Despite the fact that the basic pathogenesis of cervical cancer is settled, little is known about the molecular mechanisms underlying the malignant progression. At present, it is well‐established that cancer initiation and progression involve gradual accumulation of various genetic and epigenetic alterations that modify the transcriptional programme 3. Epigenetic changes are represented by DNA methylation, histone modifications and non‐coding RNAs activity, all having as a result genes expression regulation.

Long non‐coding RNAs (lncRNAs) consist of a recently new identified class of non‐coding RNA transcripts with the length of >200 nucleotides that have been shown to contribute to many biological processes including cellular development, differentiation and transformation 4, 5. Given the important role that these molecules play in regulation of a variety of cellular process, it is not surprising the keen interest shown by many studies that investigate their expression profile in tumorigenesis, numerous reports indicating a dysregulated lncRNAs expression in different types of human malignancies such as prostate, colorectal, hepatic, breast cancer 6, 7, 8, 9.

There are studies that emphasize an important role for these regulators in cervical carcinogenesis 10, 11. The expression of some lncRNAs has been reported to suffer important alterations in cervical cancer and has been associated with cancer progression and aggressiveness among the most notably are as follows: H19, HOTAIR, MALAT1, GAS5, MEG3, CCAT2 12, 13, 14, 15, 16, 17.

Long intergenic non‐coding RNAs (lincRNAs) are a newly described class of non‐coding RNAs that appear to be involved in a wide range of normal cellular process including differentiation, proliferation, development, cell signalling, stem cell pluripotency and lately has been displayed that several of them can control gene expression through chromatin regulation, and therefore contribute to cancer progression 18, 19, 20, 21. Despite the great knowledge accumulated so far and the high interest regarding lncRNAs involvement in various types of cancer, very little is known about lincRNAs part in oncogenesis even more in cervical cancer.

In this study, we aim to investigate the expression profile of LINC01101 and LINC00277 lincRNAs (identified through a high‐throughput method) and to explore their potential clinical significance in cervical oncogenesis.

Regarding the investigated lincRNAs, little is known about LINC00277 (EWSAT1‐Ewing sarcoma‐associated transcript 1) whereas no published data are available about LINC01101 by our knowledge.

Few studies associated LINC00277 with several types of cancer, for example this lncRNA was found to be up‐regulated and having an important role in Ewing sarcoma pathogenesis 22, while in nasopharyngeal carcinoma LINC00277, overexpression is correlated with cell growth and a poor survival rate 23. In contrast, it was reported that for non‐small cell lung cancer EWSAT1 is found to be down‐regulated in PC9 cells and appear to be involved in gefitinib resistance 24.

## Materials and methods

### Ethics statement

The current study protocol was in agreement and approved by ‘Stefan S. Nicolau’ Virology Institute Research Ethics Committee. Written informed consent was obtained from each patient prior to the study enrolment.

### Cell culture and siRNA transfection

Human cervical cancer cell line HeLa used for this study was obtained from the American Type Culture Collection (ATCC) and cells were cultured in DMEM‐F12 (1:1) medium (Gibco, Thermo Fisher Scientific Inc., Waltham, MA, USA) supplemented with 10% foetal bovine serum (FBS) at 37°C in 5% CO2 humidified incubator. For siRNA transfection HeLa cells (2.0 × 10^5^) were plated in a 6‐well dish, in antibiotic‐free growth medium supplemented with 10% FBS and cultured until 70–90% confluence. Specific siRNA for E7 HPV18 gene was designed and synthesized by Ambion (Thermo Fisher Scientific Inc.) with the following sequences: sense: 5′‐GGAAGAAAACGAUGAAAUAtt‐3′; antisense: 5′‐UAUUUCAUCGUUUUCUUCCtc‐3′. HeLa cells were transfected with siRNA‐E7HPV18 (concentration 75 nM) and siRNA control using Lipofectamine^®^ 2000 Transfection Reagent (Thermo Fisher Scientific Inc.) in Opti‐MEM™ I Reduced Serum Media (Gibco™, Thermo Fisher Scientific Inc.) according to manufacturer's recommendations. Transfection was carried out for 48 hrs and afterwards transfected cells and controls (cells cultured in transfection medium as well as untreated cells) were harvested for further studies. Transfection efficiency was evaluated according to manufacturer instructions 25 by quantifying E7 HPV18 mRNA expression levels in transfected cells and computed to be 75.4%.

### Study group

The investigated patients for this study were selected from tissue cervical specimens (*n* = 110) from women cervical lesions who self‐referred for gynaecological examinations. The samples were collected by gynaecologists and histological examination was performed by a trained pathologist. The samples were divided according to the histopathological results as follows: CIN1 (Cervical Intraepithelial Neoplasia 1) (*n* = 30, age range: 21–45 years old, median: 40.5); CIN2 (Cervical Intraepithelial Neoplasia 2) (*n* = 25, age range: 28–54 years old, median: 41); CIN3 (Cervical Intraepithelial Neoplasia 3) (*n* = 32, age range: 25–63 years old, median: 43); SCC (squamous cervical carcinoma) (*n* = 23, age range: 29–72 years old, median: 47). Also 30 cervical specimens from women (age range: 20–44 years old, median: 32) with normal biopsies and negative for HPV infection, who underwent hysterectomy for other causes than cervical cancer were included in the study as control group. Cervical samples obtained from surgical or biopsy specimens were immediately preserved in RNAlater™ Stabilization Solution (Invitrogen™, Thermo Fisher Scientific Inc.) and stored at −80°C until use.

### HPV detection and genotyping

All samples harvested from patients were tested for HPV DNA presence. DNA was isolated from tissue specimens using High Pure PCR Template Preparation Kit (Roche Molecular Biochemicals, Mannheim, Germany), according to manufacturer's recommendations. Isolated DNAs were subsequently stored at −20°C. The concentration and purity of each DNA sample were evaluated with NanoDrop ND‐1000 spectrophotometer (Thermo Fisher Scientific Inc.).

Afterwards, HPV genotyping was performed using Linear Array HPV Genotyping Test (Roche Molecular Biochemicals) according to the manufacturer's instructions. Briefly, this test uses a pool of biotinylated primers that amplify near 450 base pairs from L1 gene of 37 HPV genotypes including 13 high‐risk types (16, 18, 31, 33, 35, 39, 45, 51, 52, 56, 58, 59 and 68). An additional primer pair targets the human β‐globin gene to provide a control for cell adequacy, extraction and amplification.

### RNA isolation and cDNA synthesis

Total RNA extraction from HeLa cultured cells and tissue cervical specimens was performed using TRIzol™ Reagent (Invitrogen™, Thermo Fisher Scientific Inc.) and purified with RNeasy Mini kit (Qiagen, Hilden, Germany) according to the manufacturer's instructions. The samples were stored at −80**°**C until used. RNA quantity and quality were determined using a NanoDrop ND‐1000 spectrophotometer (Thermo Fisher Scientific Inc.) and Agilent 2100 bioanalyzer (Agilent Technologies Inc., Santa Clara, CA, USA). RNAs with a RIN (RNA integrity number) *>*7.5 were used to generate cDNAs. Synthesis of cDNA was performed using Transcriptor First Strand cDNA Synthesis Kit (Roche Molecular Biochemicals) according to the manufacturer instructions and stored at −20**°**C until further use.

### Microarray analysis

The microarray experiment was performed using One‐colour Microarray‐based Gene Expression (Agilent Technologies Inc.) according to manufacturer protocol and guidelines and scanned using Agilent Microarray Scanner System (Agilent Technologies Inc.). The obtained data were analysed with GeneSpring GX version 12 software (Agilent Technologies Inc).

### Real‐time quantitative PCR (qPCR) analysis

QPCR was performed using FastStart Universal SYBR Green Master mix (Roche Molecular Biochemicals) according to the manufacturer instructions on Applied Biosystems 7300 Real‐time PCR system (Applied Biosystems, Thermo Fisher Scientific Inc.). The expression level of each investigated gene was normalized using U6 gene as reference and each sample was analysed in duplicate. Specific primers were designed for targeted genes using primer‐BLAST (www.ncbi.nlm.nih.gov/tools/primer-blast/), and primer sequences are presented in Table 1. The qPCR data were analysed and relative expression was calculated with Cq (quantification cycle) using 2^−∆Cq^/2^−ΔΔCq^ method.

**Table 1:** List of primers sequences used in qPCR

Gene	Primer sequence
LINC00277	Forward: 5′‐CTGAAACTCCCACCGAGACC‐3′ Reverse: 5′‐ATGGGCTTAAGGGTGGGGTA‐3′
LINC01101	Forward: 5′‐GTGTCTAAGCCCCCATCACC‐3′ Reverse: 5′‐ TTTTCTTCCTGCCGAGTGGG‐3′
U6	Forward: 5′‐CTCGCTTCGGCAGCACATATACT‐3′ Reverse: 5′‐ACGCTTCACGAATTTGCGTGTC‐3′

### Western blot analysis

Total cellular proteins were extracted by disrupting cells in a lysis buffer using cOmplete™ Lysis‐M **(**Roche Molecular Biochemicals). For protein analyses, 35 μg of protein extract were separated by 12.5% SDS‐PAGE, transferred to an Immobilon‐P PVDF membrane (Merck Millipore, Billerica, MA, USA) and analysed by enhanced chemiluminescence (Thermo Fisher Scientific). The following primary antibodies were used as follows: anti‐HPV18 E7 antibody (ab100953; Abcam, Cambridge, UK) at a dilution of 1/1000 and monoclonal antibeta‐actin antibody (A5441; Sigma‐Aldrich, St. Louis, MO, USA) at a dilution of 1/1000.

### Apoptosis analysis

Apoptosis in transfected cells was evaluated in flow cytometry using FITC Annexin V Apoptosis Detection Kit I (BD Biosciences, San Jose, CA, USA). Cells were harvested 48 hrs following transfection and stained with Annexin V according to the manufacturer instructions and later analysed using BD FACSCANTO II (BD Biosciences, San Jose, CA, USA) flow cytometer.

### Statistical analysis

Statistical data were expressed as mean ± S.D. and analysed using the following software's: SPSS version 20.0 (IBM, San Jose, CA, USA) and GraphPad Prism version 5.0 (Graph Pad Software Inc., San Diego, CA, USA). For statistical analyses, one‐way ANOVA nonparametric (Kruskal–Wallis) test, nonparametric *t*‐test (Mann–Whitney), contingency analysis (Fisher's exact test) and linear regression test were used. GraphPad Prism 5.0 was used for figure drawings. *P* values <0.05 were considered statistically significant.

## Results

### Long non‐coding RNAs expression in E7HPV18 knock‐down HeLa cell line

**Figure 1:**
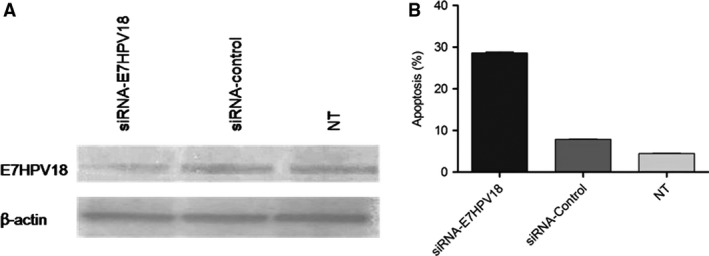
(**A**) Western blot analysis of HeLa cell line following transfection with siRNA‐E7HPV18. The cells were harvested after 48 hrs and analysed for the expression of protein levels of E7HPV18 and β‐actin as loading control. Results indicated reduced levels of E7 protein in siRNA transfected cells. (**B**) The apoptosis rate of HeLa cells was measured after transfection and the stained cells were analysed by flow cytometry and the results showed that transfection with siRNA‐E7HPV18 leads to an increase of total apoptosis up to 28.3% as compared with controls.

HeLa cell line was used as experimental model for E7HPV18 silencing with specific siRNA (75 nM). The results showed that highest knock‐down percentage was obtained 48 hrs after transfection (75.4%) in E7‐siRNA transfected cells leading to reduced expression levels of the viral oncogene (Fig. 1).

Cells transfected with E7‐siRNA were latter subjected to microarray and after analysing the obtained results, we noted that among 15387 RNA species found to be deregulated in E7‐siRNA transfected cells compared with controls (fold change >2) there were 731 lncRNAs and 203 lincRNAs.

Further on, after analysing the gathered data, it was noted that among 203 identified lincRNAs LINC00277 (EWSAT1‐ Ewing sarcoma‐associated transcript 1) and LINC01101 that were the most up‐regulated, exhibiting an increase of 15.987‐fold and, respectively, 14.962‐fold.

**Figure 2:**
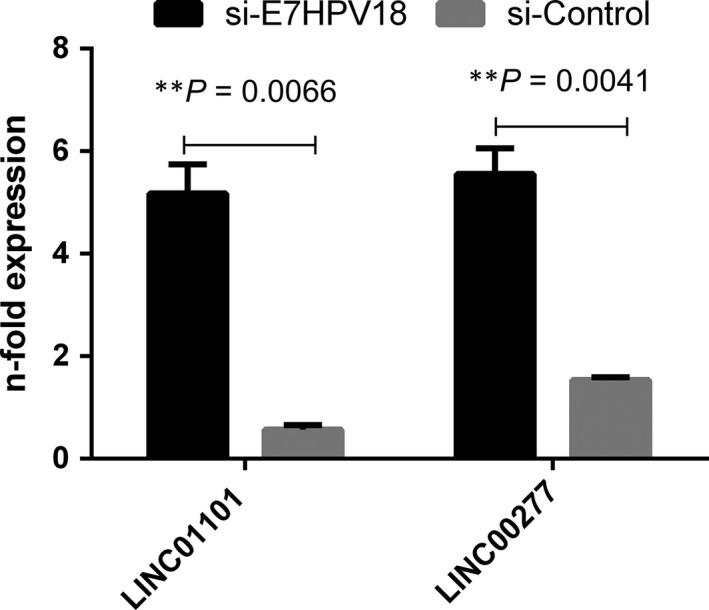
Expression levels of LINC01101 and LINC00277 genes in E7‐siRNA treated HeLa cell line. LincRNAs levels were measured by qRT‐PCR following transfection with siRNA E7HPV18. U6 gene was used as reference. Results are expressed as 2^−ΔΔCq^. All data are shown as mean ± S.D. Statistical analyse was performed using Mann–Whitney test. *P < 0.05, **P < 0.001.

Next, to validate the microarray data in case of LINC01101 and LINC00277, their levels were quantified by qRT‐PCR in cDNAs obtained following transfection with siRNA E7HPV18. The results showed a significantly increased expression levels for both LINC01101 and LINC00277 in E7‐siRNA transfected cells as compared with controls (*P* = 0.0066, respectively *P* = 0.0041; Fig. 2).

To validate the differences observed in microarray analysis, the expression of selected lincRNAs (LINC01101 and LINC00277) in precancerous and cancerous lesions from patients was assessed.

From all the 110 investigated patients in this study, after HPV genotyping analysis, 96 patients were positive for HPV infection mostly with high‐risk genotypes especially HPV16 and HPV18 detected either in single infection (66.7%) or coinfections with other genotypes (33.3%).

The distribution of HPV genotypes detected for the investigated patients depending on the pathological results it is presented in Table 2.

**Table 2:** HPV genotypes distribution according to the histopathological results^a^

HPV genotype	CIN1 (*n* = 30)	CIN2 (*n* = 25)	CIN3 (*n* = 32)	SCC (*n* = 23)
16	11 (36.7%)	10 (40%)	14 (43.7%)	8 (34.8%)
18	6 (20%)	8 (32%)	8 (25%)	10 (43.5%)
HR^b^	8 (26.6%)	4 (16%)	6 (18.8)	3 (13%)
Negative	5 (16.7%)	3 (12%)	4 (12.5%)	2 (8.7%)

a Values are presented as number (%).

b Other high‐risk types including HPV ‐31, ‐33, ‐35, ‐45, ‐51, ‐52, ‐58, ‐59, ‐ 68.

### Down‐regulation of LINC01101 and LINC00277 in precancerous and cancerous lesions

**Figure 3:**
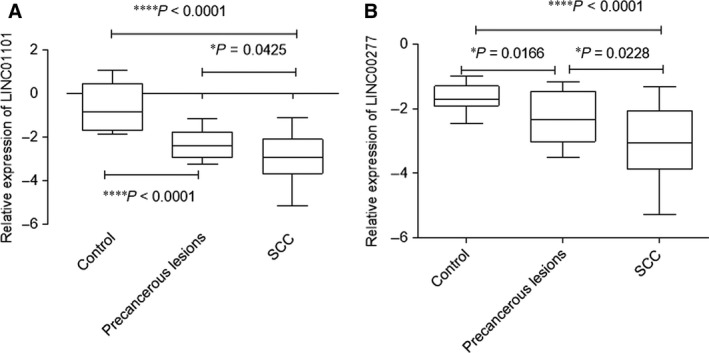
Relative expression levels of LINC01101 (A) and LINC00277 (B) in precancerous lesions and cervical cancer. All data are expressed as log10 (2^−ΔCq^). *P < 0.05, **P < 0.001, ****P < 0.0001.

Analysing the expression of LINC01101 for the investigated patients, it was found that its levels progressively decrease in precancerous lesions (mean = −2.329 ± 0.641, *P* < 0.0001) and SCC group of patients (mean = −2.970 ± 1.129, *P* < 0.0001), as comparing with the control cases (mean = −0.612 ± 1.09). Also a significant difference regarding LINC01101 levels was noted between precancerous lesions and SCC (*P* = 0.0425; Fig. 3A).

For LINC00277, a similar pattern of expression was found, the most reduced LINC00277 relative expression level was observed for SCC cases (mean = −3.049 ***±*** 1.102, *P* < 0.0001), as for precancerous lesions also decreased levels were detected (mean = −2.304 ± 0.800, *P* < 0.0001), compared with control group (mean = −1.670 ± 0.412; Fig. 3B).

Even though there is no statistical significance (*P* = 0.0753), we observed a linear correlation (*r* ^2^ = 0.7048) between LINC01101 expression level and histological grade. On the other hand, analysing a potential correlation between LINC00277 levels and histological grade, we observed a significantly (*P* = 0.0427) linear correlation (*r* ^2^ = 0.7933).

### Correlations between the expression of the investigated lincRNAs and clinical characteristics in cervical cancer

To investigate the clinical relevance for the studied lincRNAs in cervical cancer, we examined potential correlations between their expression and some clinicopathological features such as histological grade, FIGO stage and lymph node metastasis. Median expression level of LINC01101 (median = −2.923) and LINC00277 (median = −3.070) was used as a cut‐off point to divide all SCC patients into two groups: low‐expression group patients expressing lincRNAs less than the median expression level and high expression group those with the expression equal or above the median expression.

Our results showed that low expression of LINC01101 correlates significantly with FIGO stage (*P* = 0.0094), lymph node metastasis (*P* = 0.0361) but not with histological grade (Table 3). On the other hand, for LINC00277, no association between its reduced expression and clinicopathological parameters was found.

**Table 3:** Correlation between LINC01101 and LINC00277 expressions with clinical features for patients^a^ with cervical cancer

Clinical characteristic	Low *LINC01101* expression	High *LINC01101* expression	*P*‐value^b^	OR (95% CI)^b^	Low *LINC00277* expression	High *LINC00277* expression	*P*‐value^b^	OR (95% CI)^b^
Histological grade								
G1 + G2	7/16	9/16	0.3707		7/16	9/16	0.3707	
G3	5/7	2/7		0.311 (0.045–2.11)	5/7	2/7		0.311 (0.045–2.11)
FIGO stage								
I‐II	4/14	10/14	**0.0094**	0.05 (0.0046–0.54)	5/14	9/14	0.0894	0.1587 (0.0233–1.077)
III‐IV	8/9	1/9			7/9	2/9		
Lymph node metastasis								
YES	8/10	2/10		9 (1.285–63.02)	7/10	3/10		3.733 (0.645–21.58)
NO	4/13	9/13	**0.0361**		5/13	8/13	0.2138	

a Values are presented as number.

b *P*‐value, odds ratio (OR) and 95% confidence interval (CI) were calculated using Fisher's exact test.

Significant values are typed in bold.

### A reduced expression of LINC01101 and LINC00277 is associated with hrHPV infection in cervical samples

Further, in our study, we investigated a potential correlation between LINC01101 and LINC00277 expression profile and hrHPV (especially HPV16 and HPV18 genotypes) in cervical samples.

Overall the obtained results displayed a very significantly reduced expression for the two lincRNAs in hrHPV‐positive cervical samples as compared with HPV negative (*P* < 0.0001).

**Figure 4:**
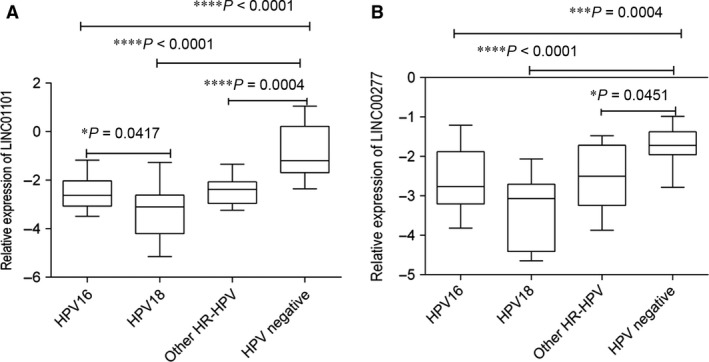
Relative expression levels of LINC01101 (**A**) and LINC00277 (B) in hrHPV‐positive (HPV16, HPV18, other high‐risk genotypes) *versus* HPV‐negative samples. All data are expressed as log10 (2^−ΔCq^)^.^ *P < 0.05, **P < 0.001, ****P < 0.0001.

We noted that for both tested lincRNAs, the lowest expression level was recorded in HPV18 infected group of patients as follows: LINC01101 (mean = −3.276 **±** 1.09) and for LINC00277 (mean = −3.362 ***±*** 0.9186). Notable LINC01101 displayed a statistical difference in expression between patients with HPV16 and HPV18 (*P* = 0.0417; Fig. 4).

Moreover, an interesting observation was made regarding LINC00277 expression levels in HPV‐positive patients with high‐risk genotypes detected in single or coinfections. For all the investigated patients, a more significantly reduced LINC00277 levels (*P* = 0.0102) were found in HPV single infections (mean = −3.178 **±** 0.877) as compared with coinfections (mean = −2.231 **±** 0.95).

**Figure 5:**
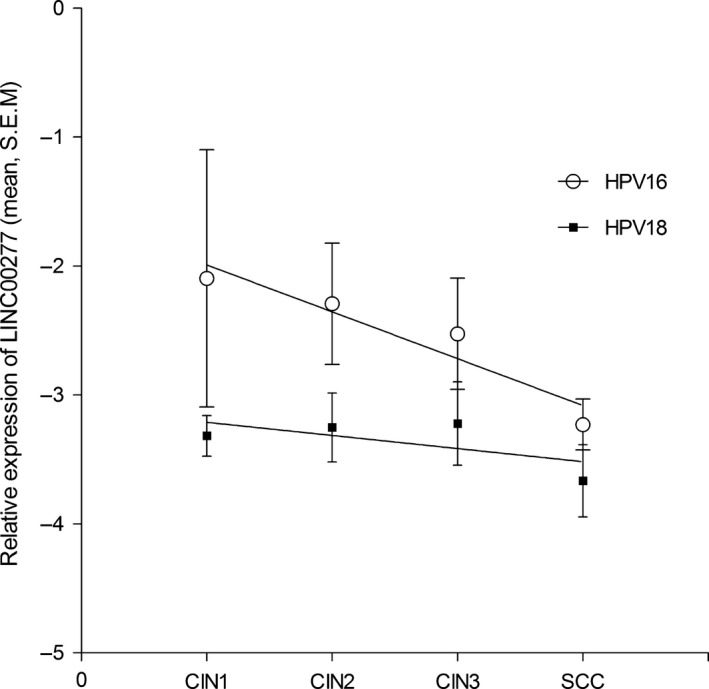
Expression levels of LINC00277 *versus* lesion severity in HPV16, HPV18‐positive samples. All data are expressed as mean ± S.E.M.

Although that the overall LINC00277 expression levels for HPV18‐positive samples were lower than for HPV16, a better correlation between decreasing LINC00277 levels and lesion severity was noted for HPV16 genotype (slope: −0.3632 **±** 0.08569; *r* ^2^ = 0.899; *P* = 0.05; Fig. 5) as in contrast to HPV18 infections where it was also observed a decrease but without statistically significance.

Partial correlation analysis between LINC00277 and LINC01101 values as variable with controls variables CIN grade and HPV presence showed that both studied lincRNAs have an influence on the status [moderate for CIN r(18) = 0.557, *P* = 0.011, and high for HPV r(18) = 0.596, *P* = 0.006].

## Discussion

Regardless of the worldwide implementation of cervical screening programmes and the advanced therapeutic strategies, cervical cancer remains the fourth cause of cancer death in women and a major public health problem 1, 26. Currently, there are ongoing efforts to find new factors that contribute to the development and progression of this malignancy and that could serve as novel markers for early detection and prognosis.

In the recent years, an attractive direction in cervical cancer research has been studying the epigenetic changes that occur in malignant transformation and the roles that they play. Numerous studies revealed the potential role of lncRNAs molecules in cervical cancer progression, invasion and metastasis. For example, reports have shown that lncRNA HOTAIR is significantly overexpressed in cervical cancer and furthermore its expression is correlated with tumour progression and metastasis and can predict a poor prognosis in patients with cervical cancer 13, 27, 28. Another intensely studied lncRNAs in cervical oncogenesis is MALAT1 who was found to be highly expressed in cancer advances stages, shown to promote proliferation, growth and invasion and overall can predict a poor prognosis 29, 30. Moreover, MALAT1 deregulated expression in cervical cancer has been link with high‐risk genotypes HPV infection 14. There are also some lncRNAs whose low expression is associated with cervical cancer progression and poor prognosis and such examples are lncRNA LET and XLOC _010588 31, 32.

Taken into consideration all the gathering data that points out the important role played by lncRNAs in cervical oncogenesis, in the current study, we decided to evaluate the expression pattern of two lincRNAs in cervical lesions and cancer, to test their clinical significance and the connection with hrHPV infection.

Our data indicate that both lincRNAs display a significantly reduced pattern of expression in cervical lesions and cancer. A significantly lower level of LINC01101 and LINC00277 is present in advanced stages of cervical cancer. Moreover, for patients with cervical cancer after testing the clinical relevance for the two lincRNAs, we found that LINC01101 markedly down‐regulated expression is significantly correlated with FIGO stage and lymph node metastasis. Taken together, these findings suggest that for patients with squamous cervical carcinoma LINC01101 relative expression may serve as a potential prognostic factor.

On another hand, in the present study a connection between HPV presence and lincRNAs it was highlighted. The samples positive for high‐risk HPV infection exhibit a notable reduced LINC01101 and LINC00277 expression level than those HPV negative (*P* < 0.05).

Data showed that from the two investigated lincRNAs, LINC00277 appears to correlate better with HPV infection asit was found that its significantly reduced levels were detected in single infections with HPV high‐risk genotypes and also that in case of infections with HPV16 LINC00277 decreased levels correlate with lesion severity.

To the best of our knowledge, this is the first study investigating LINC01101 and LINC00277 expression levels in cervical oncogenesis.

In conclusion, our study has demonstrated that expression levels of novel intergenic lncRNAs: LINC01101 and LINC00277 are down‐regulated in cervical cancer and that they are associated with progression and high‐risk HPV infection. These findings provide new insights into lncRNA involvement in HPV‐induced cervical cancer and indicate a potential role for this factors in cervical cancer development suggesting also a potential role for prognosis and target for cervical cancer therapies, but further studies are needed to establish the potential role for studied lncRNAs in this pathology.

## Conflict of interest

The authors confirm that there are no conflicts of interest.
